# High genetic variability of HIV-1 in female sex workers from Argentina

**DOI:** 10.1186/1742-4690-4-58

**Published:** 2007-08-13

**Authors:** María A Pando, Lindsay M Eyzaguirre, Gladys Carrion, Silvia M Montano, José L Sanchez, Jean K Carr, María M Avila

**Affiliations:** 1 Centro Nacional de Referencia para el SIDA (CNRS), Departamento de Microbiología, Parasitología e Inmunología, Facultad de Medicina, Universidad de Buenos Aires, Paraguay 2155, Piso 11, C1121ABG, Buenos Aires, Argentina; 2 Department of Epidemiology, Institute of Human Virology, University of Maryland Biotechnology Institute, 725 West Lombard Street, Baltimore, MD 21201, USA; 3 US Naval Medical Research Center Detachment (NMRCD). Unit 3800, APO-AA 34031-3800, Lima, Peru; 4 Department of Defense Global Emerging Infections Surveillance and Response System (DoD-GEIS), Walter Reed Army Institute of Research, 2900 Linden Lane, Suite 100, Silver Spring, MD, 20910, USA; 5 CONICET, Argentina

## Abstract

**Background:**

A cross-sectional study on 625 Female Sex Workers (FSWs) was conducted between 2000 and 2002 in 6 cities in Argentina. This study describes the genetic diversity and the resistance profile of the HIV-infected subjects.

**Results:**

Seventeen samples from HIV positive FSWs were genotyped by *env *HMA, showing the presence of 9 subtype F, 6 subtype B and 2 subtype C. Sequence analysis of the protease/RT region on 16 of these showed that 10 were BF recombinants, three were subtype B, two were subtype C, and one sample presented a dual infection with subtype B and a BF recombinant. Full-length genomes of five of the protease/RT BF recombinants were also sequenced, showing that three of them were CRF12_BF. One FSW had a dual HIV-1 infection with subtype B and a BF recombinant. The B sections of the BF recombinant clustered closely with the pure B sequence isolated from the same patient. Major resistance mutations to antiretroviral drugs were found in 3 of 16 (18.8%) strains.

**Conclusion:**

The genetic diversity of HIV strains among FSWs in Argentina was extensive; about three-quarters of the samples were infected with diverse BF recombinants, near twenty percent had primary ART resistance and one sample presented a dual infection. Heterosexual transmission of genetically diverse, drug resistant strains among FSWs and their clients represents an important and underestimated threat, in Argentina.

## Background

The genetic diversity of human immunodeficiency virus type 1 (HIV-1) was recognized early in the epidemic. Phylogenetic analyses of HIV-1 have revealed the presence of 9 subtypes (A-D, F-H, J and K) and at least 34 circulating recombinant forms (CRFs) worldwide. A great variety of unique recombinant structures have also been identified [1]. Previous studies have highlighted the complex nature of the HIV epidemic in Argentina and revealed the presence of two independent epidemics: one among men who have sex with men (MSM) where the viral strains are mostly subtype B, and the second among heterosexuals and injecting drug users (IDUs) where BF recombinants predominate [2,3]. Further sequencing studies have revealed the presence of a new CRF, CRF12_BF [4]. More recently, phylogenetic analysis of strains from Argentina has described different subtypes and recombinants in newly diagnosed patients [5], including one triple recombinant between subtypes B, C and F [6].

Female sex workers (FSWs) have been at high-risk of infection since the beginning of the HIV epidemic all over the world. Multiple sex partners, irregular condom use, and co-infection with other sexual transmitted infections (STIs) are the principal risk factors for HIV infection among FSWs [7]. During March 2000 and March 2002, a cross-sectional seroprevalence study was conducted among 625 FSWs in six cities in Argentina (Buenos Aires, Salta, Rosario, Córdoba, Mendoza and La Plata) with the objective of estimating the HIV prevalence and associated risk factors [8]. The findings of this study clearly indicated that this population of FSWs was found to be at high risk of STIs, as illustrated by the high prevalence found for syphilis (45.7%), hepatitis B (14.4%), HIV (3.2%), hepatitis C (4.3%), and HTLV-I/II (1.6%) infections. The study also showed that this group has sexual contact with men from other countries: 51.2% of them reported having had sexual contact with foreigners, mostly from Brazil and Paraguay. In addition, FSWs have multiple exposures due to the number of sex partners they have over time (mean per week: 14).

The main objective of this retrospective study was to describe the genetic diversity and antiretroviral resistance profile of HIV strains among Argentinean FSWs who represent an important core group, which can serve as a link with subsequent transmission to the heterosexually active population at-large.

## Methods

### Study population

Details of the enrollment and data collection procedures for this study have been reported previously [8]. FSWs (n = 625) were recruited through a non-governmental organization (AMMAR: Asociacion de Mujeres Meretrices de Argentina) for a cross-sectional seroprevalence study. Confidential one-on-one interviews were conducted on-site by health care workers. During these encounters, the study was explained and subjects were invited to participate. Only those subjects who were willing to participate were provided written informed consent, enrolled, and sampled. Study participants were interviewed using a standardized questionnaire with information regarding sociodemographic characteristics, sexual practices, current or past use of illegal drugs, and prior history of STIs. FSWs declared not to be under HIV antiretroviral treatment.

This research was reviewed by the institutional review boards and scientific ethical committees at the University of Buenos Aires and at the U.S. Naval Medical Research Center (NMRC) in the United States and was conducted in compliance with all federal regulations governing the protection of human subjects.

### Blood sampling and genotyping procedures

Peripheral blood mononuclear cells (PBMCs) from HIV-infected FSWs recruited during the seroprevalence study were obtained, separated by Ficoll-Hypaque technique and maintained at -70°C. PBMCs were used for DNA extraction by the QIAmp DNA extraction kit (QIAgen, Valencia, CA, USA). All 17 samples were subjected to PCR amplification in *env*, 16 were amplified at the protease/reverse transcriptase (pro/RT) region, and 6 samples were subjected to near full-length amplification. The *env *region was amplified using ED3/ED14 as outer primers and ED31/ED33 as inner primers. Heteroduplex mobility assay (HMA) was performed with the second-round PCR products using nine reference standards as previously described [9]. The pro/RT region was amplified using Por5F/RT3474R in the first round and Pro3F/ProRT for the second round. The conditions of the PCR amplification have been previously described [10].

Near full-length sequences were done with primers MSF12b and OFMR1 for the first-round. The second-round amplification was completed using 1 μl of the first-round product and primers F2NST and UNINEF 7. This nested strategy amplifies about 9000 kb of the HIV genome and was slightly modified from that used previously [11,12]. The amplified products were then sequenced with Big Dye terminators using an ABI 3100 automated sequencer (Applied Biosystems Inc, Foster City CA).

Phylogenetic analysis was performed by first aligning the sequences obtained with reference sequences using the program MacGDE [13] followed by a Neighbor-joining method with Kimura's two-parameter model of distance calculation; bootstrap analysis was performed with 100 replicas. Phylogenetic trees were constructed using MEGA3 [14,15]. Recombinant analysis was performed using SimPlot v.3.5.1 [16].

### Resistance profile

The genotypic antiretroviral drug resistance profile was examined by examining for mayor mutations which have been associated with reduced susceptibility to protease and RT inhibitors, as reported by the International AIDS Society-USA in April 2005 (Stanford University HIV Drug Resistance Database). RT: M41L, A62V, K65R, D67N, T69D, 69 insert, K70R, L74V, V75I, F77L, L100I, K103N, V106A, V106M, V108I, Y115F, F116Y, Q151M, Y181C, Y181I, M184V, M184I, Y188C, Y188L, Y188H, G190A, G190S, L210W, T215Y, T215F, K219Q, K219E, P225H, M230L, and P236L; protease: D30N, M46I, M46L, G48V, I50V, V82A, V82S, V82F, V82T, I84V, and L90M.

## Results

### Characteristics of the study participants

A total of 625 FSWs were enrolled in the seroprevalence study and 20 tested HIV positive, reflecting a prevalence of 3.2% [8]. These HIV positive samples were from different cities in the country, one from La Plata (1/100, 1.0%), two from Cordoba (2/86, 2.3%), two from Mendoza (2/33, 6.1%), five from Salta (5/98, 5.1%) and ten from Buenos Aires (10/296, 3.4%). No infections were detected in Rosario (0/12)

### Genetic Characterization

#### Heteroduplex Mobility Assay (HMA)

Out of 20 HIV positive samples, envelope HMA was completed in 17 samples. As shown in Table 1, env subtype F was found in 52.9% (n = 9), subtype B in 35.3% (n = 6) and subtype C in 11.8% (n = 2).

**Table 1:** HIV subtype by heteroduplex mobility assay (HMA), Protease/RT sequence and nearly full length sequence and major resistance mutations in FSWs, Argentina, 2000–2002.

Sample ID	City	HMA	ProRT	Full length	Major resistance mutations
00AR1034	Buenos Aires	B	B	ND	No Detected
02AR4013	Mendoza	B	B	ND	L74V – K103N
02AR4014	Mendoza	B	B	ND	M41L
00AR2141	Buenos Aires	B	ND	ND	ND
01AR4008	Cordoba	B	**B/BF Dual infection**	**B/BF Dual infection**	No Detected
00AR2140	Buenos Aires	B	BF	BF (URF)	No Detected
00AR1120	Buenos Aires	F	BF	ND	No Detected
00AR2002	Buenos Aires	F	BF	**CRF12_BF**	No Detected
00AR2003	Buenos Aires	F	BF	**CRF12_BF**	No Detected
00AR2004	Buenos Aires	F	BF	ND	No Detected
00AR2005	Buenos Aires	F	BF	**CRF12_BF**	No Detected
00AR2006	Buenos Aires	F	BF	ND	No Detected
01AR4001	Cordoba	F	BF	ND	No Detected
01AR4003	Salta	F	BF	BF (URF)	V108I
02AR4010	Salta	F	BF	ND	No Detected
01AR4005	La Plata	C	C	ND	No Detected
02AR4009	Salta	C	C	ND	No Detected

ND: no done. Minor mutations are described in the text.

### Sequencing analysis

**Figure 1:**
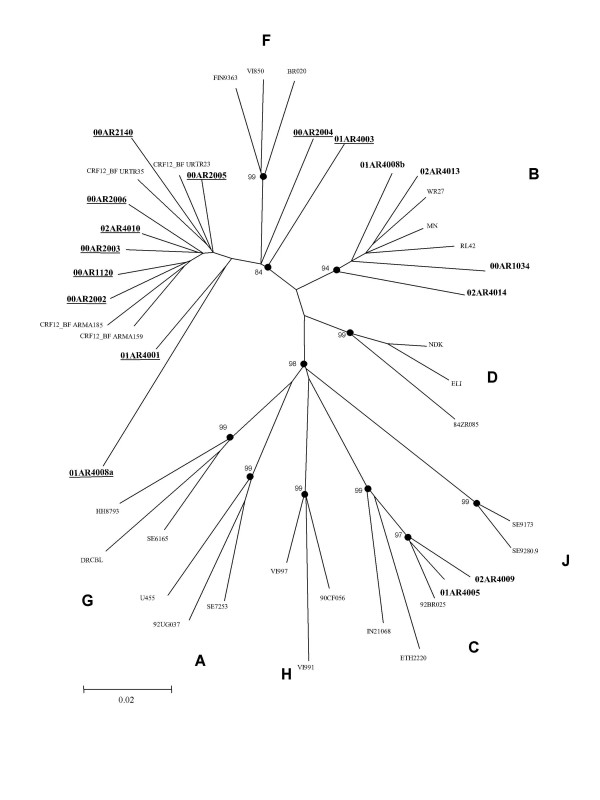
Phylogenetic analysis of 16 pol sequences from FSWs from different cities of Argentina. A neighbor-joining phylogenetic tree analysis was performed with the Kimura two-parameter method of distance estimation using reference sequences. The genetic distance corresponding to the lengths of the branches is shown by the bottom line. Studied samples are in bold. Underlined samples are inter-subtype recombinants. Sample 01AR4008a and 01AR4008b belong to the same patient.

Sequencing analysis of the pro/RT region was performed in 16 of the samples (there was no sufficient pro/RT gen amplified product in one sample). Phylogenetic analysis of these samples is shown in Figure 1. Ten samples (62.5%) were found to be BF recombinants, three were subtype B, two were subtype C, and one sample presented a dual infection with subtype B and a BF recombinant.

**Figure 2:**
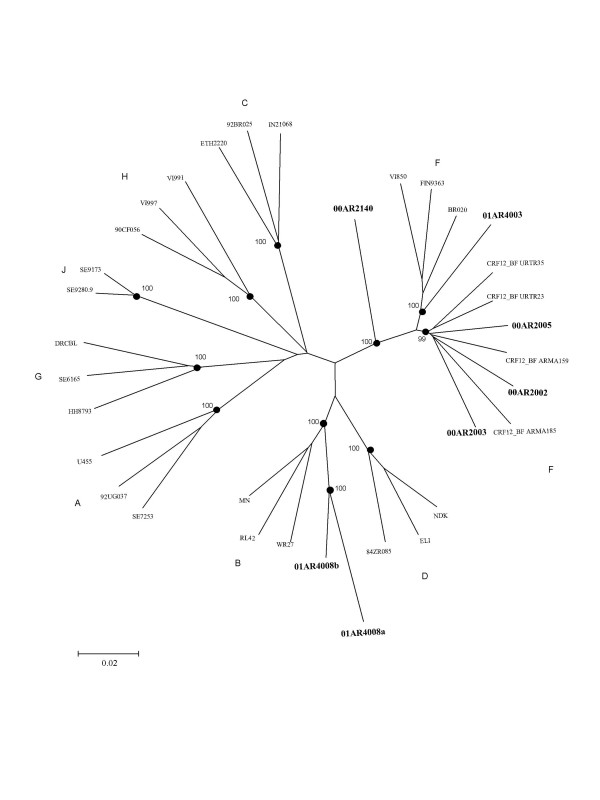
Phylogenetic analysis of 7 nearly full length sequences from FSWs from different cities of Argentina. A neighbor-joining phylogenetic tree analysis was performed with the Kimura two-parameter method of distance estimation using reference sequences. The genetic distance corresponding to the lengths of the branches is shown by the bottom line. Studied samples are in bold. Underlined samples are inter-subtype recombinants. Sample 01AR4008a and 01AR4008b belong to the same patient.

All of the samples that were characterized as subtype F (n = 9) by envelope HMA, were BF recombinants in the pro/RT gen region (Table 1). The two samples classified as subtype C by HMA were also subtype C in pro/RT gen region. Of the samples that were characterized as subtype B by envelope HMA (n = 6), 3 were subtype B in pro/RT and one was a BF recombinant. The near full-length genomes of 5 of the pro/RT BF recombinants were also sequenced (Figure 2). Simplot and Bootscan analysis of these 5 samples showed that 3 of them were CRF12_BF and 2 were unique recombinant forms (URFs, data not shown). The 2 subtype C strains significantly clustered with subtype C from Brazil.

### Double Infection (sample 01AR4008TS)

**Figure 3:**
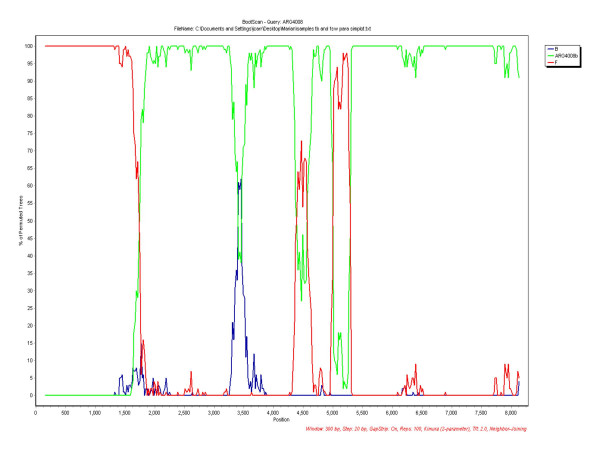
Bootscan of the BF full length HIV-1 sequence from patient 01AR4008. Consensus sequences of subtype B and F, as well as subtype B sequence of the same patient (01AR4008b) were used for comparison.

Participant 01AR4008TS was discovered by chance to have a dual infection. This sample was characterized as subtype B by envelope HMA and subsequently the pro/RT region was amplified and the sequence was characterized as a BF recombinant. The sample was then amplified to obtain a near full-length genome to corroborate the results obtained from the pro/RT, however, the amplicon resulting was non-recombinant subtype B. In order to exclude possible contamination or mixing of samples, the near full-length PCR was repeated; obtaining a BF recombinant that matched the BF pro/RT previously obtained. Simplot and Bootscan analysis of the BF recombinant was performed with a B consensus with reference samples and the B strain found in the same patient. The results obtained showed that the B regions of the BF recombinant were closer to the strain found in the same patient than to the subtype B consensus (Figure 3). The same result was obtained when consensus sequence of subtype B was performed with samples from Argentina.

**Figure 4:**
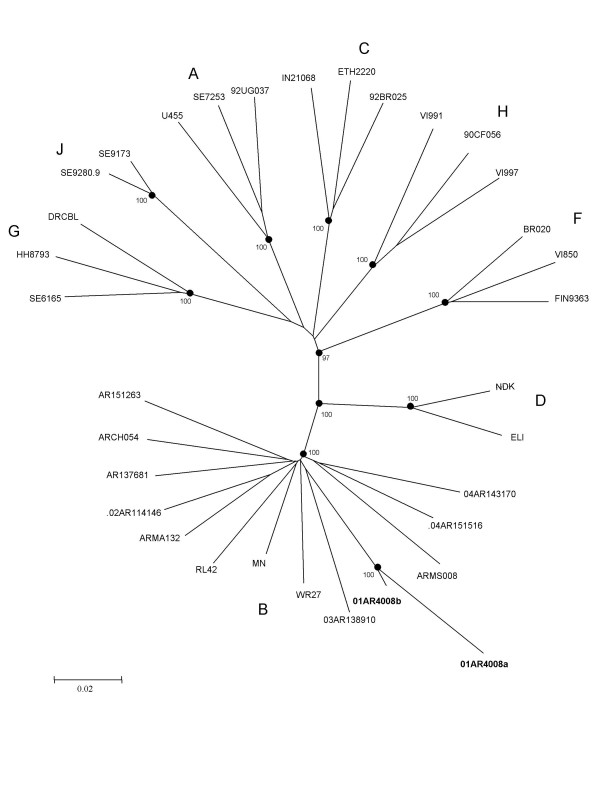
Phylogenetic analysis of concatenated B parts of the BF recombinant sample (01AR4008a) of the patient 01AR4008 with the subtype B sample from the same patient (01AR4008b), other subtype B samples from Argentina and the reference samples. A neighbor-joining phylogenetic tree analysis was performed with the Kimura two-parameter method of distance estimation using reference sequences. The genetic distance corresponding to the lengths of the branches is shown by the bottom line. Studied samples are in bold.

Phylogenetic analysis concatenating the B parts of the full length BF recombinant and using other samples from Argentina, showed that the B region of the BF recombinant and the subtype B isolate were closely related, with a bootstrap value of 100% (Figure 4). This FSW was in her twenties, and unmarried. She reported having been a sex worker for 8 years, with an average of 12 clients per week, and reported irregular use of condoms with clients. She was diagnosed with a co-infection with *Treponema pallidum *and had no past history of illegal drug use.

### Resistance Profile

Primary anti-retroviral resistance to reverse transcriptase inhibitors was detected in 3 patients out of 16 studied, leading to a rate of primary resistance of 18.8% (Table 1). The major resistance mutations detected were: L74V, K103N, M41L and V108I. L74V causes resistance to DDI and ABC. K103N causes multi NNRTI resistance and M41L multi NRTI resistance. Mutation V108I causes resistance to NVP and EFV. Secondary mutations were detected in samples 02AR4014 (T215N) and 00AR2140 (V179E).

## Conclusion

This study shows, for the first time in Argentina, the genetic variability of the HIV-1 viral strains circulating among FSWs from different cities in Argentina. As stated previously, Argentina has two predominant epidemics; subtype B among men who have sex with men (MSM) and subtype BF among IDUs and the heterosexually-active population [2,3]. It is only recently that other subtypes such as the CRF12_BF and subtype C have been identified in Argentina. The presence of subtype C has been reported previously among heterosexuals in Argentina and in most countries of the South American region [17]. However, subtype C is the most prevalent HIV subtype in the global epidemic and it is also becoming important in the Brazilian epidemic [18]. In this study, phylogenetic analysis showed that the two subtype C samples found grouped with the consensus sample from Brazil. This suggests that subtype C has been recently introduced to Argentina and it was possibly through Brazil. The potential evolution of subtype C in the Argentinean epidemic will require future molecular epidemiologic studies among heterosexually-active populations in this country.

The co-existence of subtype B and BF recombinants in the population appears to be consistent with previous reports in heterosexual women [2]. However, this is the first report of a dual HIV infection with subtype B and a BF recombinant where the BF recombinant shares the B section with the B sequence isolated from the same patient. For recombination to occur between distinct HIV strains, a single cell needs to be infected with different viruses, both of which must integrate their genome into the cell genome. The cell needs to transcribe the integrated genome of both viruses and in the packaging process a heterodimeric virus can be formed. This virus can then infect another cell and recombination between the two viral RNAs can occur during the reverse transcription process [19]. This dual infection may occur during the primary infection or it may occur as a re-infection with a new viral strain after the initial strain has established a chronic infection. It is unknown if the double infection detected in this subject (01AR4008) is the result of a dual infection at the primary infection or if it is the result of a re-infection. It can be hypothesized, however, that the BF recombinant originated through a recombination between the B strain presented in the patient and an F subtype or another BF recombinant not detected. Likewise, the B strain could have been generated from the B portion of a BF recombining with a B strain.

A similar case has been reported in the Nairobi sex worker cohort [20]. In this case, one woman had evidence of both superinfection and recombination. A first sequence of the patient was entirely subtype A, and a new sequence obtained 10 years later was A/C recombinant with a SimPlot demonstrating that the subtype A fragment in the AC recombinant was derived from the original A subtype sequence. As this study has two different time points, and they use HTA (Heteroduplex Tracking Assay) to demonstrate that the subtype C sequence was not detectable as minor species in the first sample, they can almost prove that the event of intersubtype recombination resulted from superinfection intra patient.

This study was not initially designed to detect dual infections, no follow-up was performed, and only one sample of each FSW was analyzed. This limits the conclusion that can be made, it is not known if these two related strains originated in this patient or if they were acquired from another person. It is important to point out that the identification of this dual infection was a chance event, there was not systematic search for dual infections in the other samples.

Most of the FSWs reported having had sexual relationships with multiple partners and thus were potentially exposed to many HIV variants. It is important to see not only the subtype distribution circulating in this group and the event of the dual infection, but also the resistance profile that is circulating among them since this epidemic can potentially spread the virus to the general population. The presence of resistant viruses in this group should cause concern to the public health community. Previous surveys performed in Argentina reported prevalences of mutations between 7.7% [21] and 15.4% [22] in drug-naïve patients and individuals with primary infection, respectively. The prevalence detected in our study shows to be higher that the previously reported. However, this study does not encompass the minimum number of patient (n = 52) recommended by the World Health Organization for HIV drug resistance survey [23].

Finally, infection with two HIV strains is well documented and the large number of intersubtype recombinants identified shows that dual infections must be a common occurrence. The frequency and clinical consequences of co-infection, superinfection and recombination are unknown. However, some reports have associated the dual infection with rapid disease progression [24]. For this reason, HIV-infected FSWs should be warned that safe-sex practices are still necessary to prevent superinfections and associated disease progression.

## Abbreviations

FSWs: Female sex workers

HIV-1: Human immunodeficiency virus type 1

CRFs: circulating recombinant forms

MSM: men who have sex with men

IDUs: injecting drug users

STIs: sexual transmitted infections

PBMCs: peripheral blood mononuclear cells

HMA: heteroduplex mobility assay

URFs: unique recombinant forms

NNRTIs: Non-Nucleoside Reverse Transcriptase Inhibitors

HTA: Heteroduplex Tracking Assay

## Nucleotide accession number

GenBank accession numbers for the sequences of this study are in progress.

## Competing interests

The author(s) declare that they have no competing interests.

## Authors' contributions

MAP coordinated the recruitment of the participants, did the data analysis of the sequence, and wrote the first draft of the manuscript. LME and GC help with the laboratory work. SMM and JLS made the international coordination of the study. JKC coordinated the work at the sequencing laboratory. MMA designed and directed the study. All the authors have read and approved the manuscript.
